# Switchable and Tunable Terahertz Metamaterial Based on Vanadium Dioxide and Photosensitive Silicon

**DOI:** 10.3390/nano13142144

**Published:** 2023-07-24

**Authors:** Xin Zhang, Guan Wang, Jia Liu, Shiyi Zuo, Meichen Li, Shuang Yang, Yang Jia, Yachen Gao

**Affiliations:** 1 Electronic Engineering College, Heilongjiang University, Harbin 150080, China; hi400304@163.com (X.Z.); wang2687220886@163.com (G.W.); liu_jia1220@163.com (J.L.); 18846092825@163.com (S.Z.); lmchen_qwe@163.com (M.L.); slby2025@163.com (S.Y.); jiayang_1990@163.com (Y.J.); 2 College of Communication and Electronic Engineering, Qiqihar University, Qiqihar 161000, China

**Keywords:** switchable, tunable, THz, electromagnetically induced transparency, absorber

## Abstract

A switchable and tunable terahertz (THz) metamaterial based on photosensitive silicon and Vanadium dioxide (VO_2_) was proposed. By using a finite-difference time-domain (FDTD) method, the transmission and reflective properties of the metamaterial were investigated theoretically. The results imply that the metamaterial can realize a dual electromagnetically induced transparency (EIT) or two narrow-band absorptions depending on the temperature of the VO_2_. Additionally, the magnitude of the EIT and two narrow-band absorptions can be tuned by varying the conductivity of photosensitive silicon (PSi) via pumping light. Correspondingly, the slow-light effect accompanying the EIT can also be adjusted.

## 1. Introduction

Terahertz waves are electromagnetic waves with frequencies in the range of 0.1 THz to 100 THz and wavelengths in the range of 3 μm to 3 mm, between microwave and infrared. THz technology has great potential for applications in sensing, communication, and imaging [1,2,3]. However, its development has been hampered by the limitations of natural materials. In recent years, with the advanced development of micro- and nanoprocessing technology [4,5,6], metamaterials with complex structures and increasingly small dimensions are also contributing to the rapid development of THz technology. Using metamaterials [7], researchers have designed various efficient optical micro–nano devices such as absorbers [8,9,10], polarization converters [11,12], and electromagnetically induced transparency (EIT) devices [13,14,15]. The EIT effect results from its high transmission peaks in the low-transmission region. Large dispersion and group delay effects occur near the peak frequency of the EIT transmission peak. The effect has very promising applications in the field of slow light, promotes the enhancement of optical non-linear effects, and has an equally positive effect on the development of all-optical information devices such as optical caches, optical switches, and optical routers [16,17,18,19]. The absorption effect in terahertz metamaterials has promising applications in areas such as filters, stealth devices, and highly sensitive sensors [9,20]. However, conventional THz metamaterials cannot change their properties after they are fabricated and have a single function, which leads to high practical costs, which means tunable and switchable multifunctional terahertz metamaterials are necessary.

At present, the tuning of THz metamaterials is mainly based on active materials such as VO_2_, PSi, etc. [21]. VO_2_ is a transition-metal oxide with the property of reversible phase transition. The phase transition temperature is about 340 K, which makes the phase transition of VO_2_ easy to control [21,22,23,24]. PSi is a semiconductor whose conductivity can be changed easily by tuning the intensity of light on it [25]. In 2012, J. Gu et al. combined photoconductive silicon substrates with planar metamaterial units to achieve a THz EIT effect with photocontrolled configurability [26]. In 2016, Zhang et al. proposed a dynamically modulated THz absorber combining VO_2_ films with arrays of structured copper rings. The absorber achieved a modulation depth of approximately 78% under light induction [27]. In 2018, Liu et al. introduced a dynamically tunable THz metamaterial consisting of graphene and two cut-line metal resonance rings. They realized an EIT effect with an 81% modulation depth and an actively controlled slow-light effect [28]. In 2020, Zheng et al. designed a tunable EIT terahertz metamaterial consisting of two Au split-disk resonant rings on top and bottom, with a modulation depth of 75.58% [29]. As can be seen, the studies above concerning THz metamaterials focused mainly on single functions, and had lower modulation depths. In fact, it is necessary to develop new THz metamaterials with tunable multifunctions and high modulation depths.

In this paper, we design a switchable THz metamaterial with a tunable EIT effect and absorption based on PSi and VO_2_. The metamaterial consists of metal bars and two square resonant rings of different sizes forming a metal layer, a dielectric layer, and a VO_2_ film. The properties of the designed metamaterial are investigated theoretically by means of the FDTD method. It is found that when the temperature of VO_2_ is set to be room temperature it is in the dielectric state, and the metamaterial exhibits a double EIT effect and can achieve a double slow-light effect. When the temperatures are above the phase transition temperature, VO_2_ is in a metal state and the metamaterial shows a double narrow-band absorption. When the conductivity of the PSi changes, the magnitude of EIT and absorption can be changed and both have a high modulation depth. The structure designed in this paper can be used in devices such as bio-detectors, sensors, optical switches, etc., in the corresponding frequency range [30].

## 2. Materials and Methods

**Figure 1:**
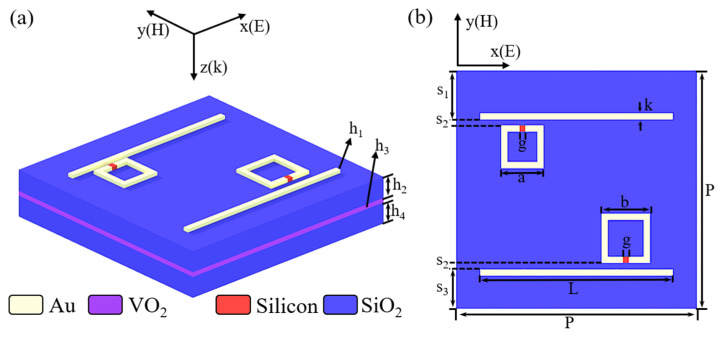
(**a**) Unit structure; (**b**) top view of unit structure.

The designed metamaterial is arranged in a periodic pattern unit with P = 200 μm. As shown in Figure 1a, from top to bottom, the unit consists of four layers which are metal layer, dielectric layer (SiO_2_), VO_2_ film, and dielectric layer (SiO_2_). Correspondingly, the thickness of each layer is h_1_ = 3 μm, h_2_ = 25 μm, h_3_ = 0.2 μm, and h_4_ = 14.8 μm, respectively. As shown in Figure 1b, the metal layer consists of metal bar (MB), large resonant ring (LRR), and small resonant ring (SRR). The length of MB is L = 160 μm, and both SRR and LRR are square rings with side lengths of a = 30 μm and b = 35 μm, respectively. The width of MB and ring is k = 5 μm. Each ring has a gap filled with g = 5 μm PSi. The distribution of each structure is given by S_1_ = 47.5 μm, S_2_ = 5 μm, and S_3_ = 37.5 μm.

The properties of the metamaterial were investigated using FDTD. When conducting simulation, the THz plane wave propagates in the Z-axis direction and its polarization is in the X-axis direction. In THz band, gold is considered to be a lossy metal, and its conductivity can be obtained as 4.56 × 10^7^ S/m via a static model [31]. The relative dielectric constants of SiO_2_ and PSi are 2.9 and 11.9, respectively [32].

The optical properties of VO_2_ are illustrated using the Drude model [33,34,35,36,37]:

(1) $$\varepsilon \left(\omega \right)=\varepsilon_{\infty }-\frac{\omega_{p}^{2}\left(\sigma \right)}{\omega^{2}+i\gamma \omega }$$

where $\varepsilon_{\infty }=12$ is the dielectric constant of VO_2_ at infinite frequency, and $\gamma =5.75\times 10^{13}$ rad/s is collision frequency [35]. The plasma frequency can be characterized as $\omega_{p}^{2}(\sigma )=\omega_{p}^{2}(\sigma_{0})\sigma /\sigma_{0}$, where $\omega_{p}\left(\sigma_{0}\right)=1.4\times 10^{15}$ rad/s, $\sigma_{0}=3\times 10^{5}$ S/m [36]. The conductivity of VO_2_ is 1 S/m or 2 × 10^5^ S/m when it is insulating or in metal phase.

**Figure 2:**
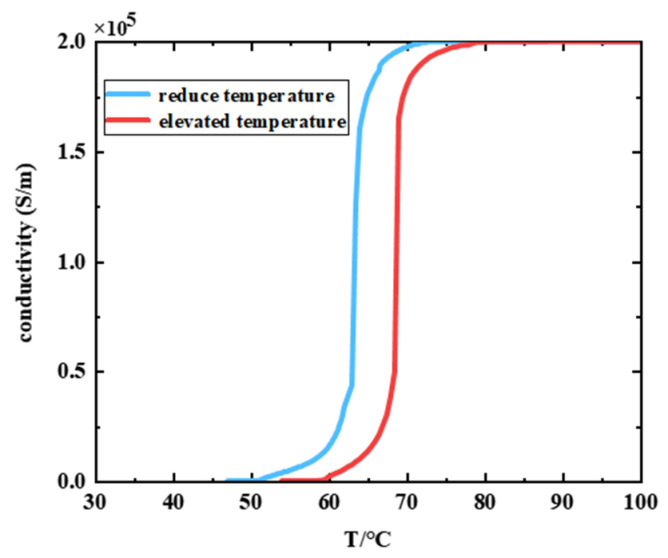
Conductivity of VO_2_ as a function of temperature.

Figure 2 shows the change in conductivity of VO_2_ with temperature, which was controlled by using electrical heating and a semiconductor refrigeration sheet. Heating from absorbed radiation will have a certain effect on the absorption characteristics of the absorber, but it is weak and can be neglected [38]. And the physical properties of Au, SiO_2_, and PSi remain essentially unchanged over the temperature range of the VO_2_ phase transition [38,39].

The conductivity of the PSi can be controlled by light and expressed as follows [40]:

(2) $$\sigma_{si}=4.863\times 10^{-4}\times I^{2}+0.1856\times I+1.569$$

where *I* is the intensity of the pump light. The conductivity of the PSi is 1 S/m and 1 × 10^5^ S/m when the intensity of the pump light is 0 or 249 μJ/cm^2^, respectively [25]. VO_2_ can also be excited by pump light, but its excitation intensity is 8 mJ/cm^2^, which is much greater than the excitation intensity of PSi [41], so the excitation of PSi with the pump light has almost no effect on VO_2_.

## 3. Results and Discussion

### 3.1. Electromagnetically Induced Transparency

**Figure 3:**
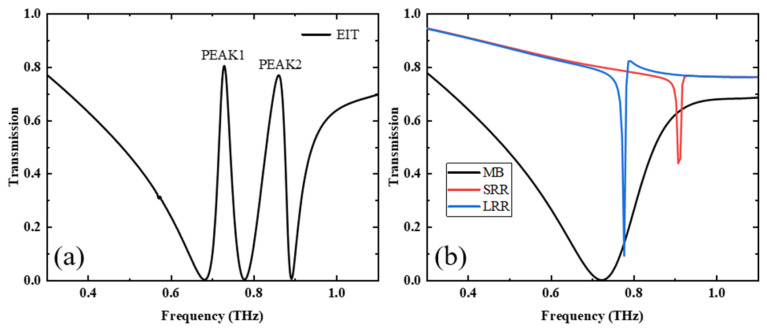
(**a**) Transmission spectra of structures consisting of MBs, SRRs, and LRRs at a conductivity of 1 S/m for both PSi and VO_2_, (**b**) transmission spectra of structures of MB, SRR, and LRR.

Firstly, we studied theoretically the transmission properties of the metamaterial when VO_2_ is below the phase-change temperature and the pumping light of the PSi is off. In this case, the conductivity of VO_2_ (25 °C) and PSi is 1 S/m [42], and the results are shown in Figure 3a. We can see that there are two transmission peaks at 0.73 THz and 0.862 THz, defined as PEAK 1 and PEAK 2, respectively. In order to investigate the origin of the transmission peaks, we individually calculated the transmissions of the MB array, the SRR array, and the LRR array, which are shown in Figure 3b using black, red, and blue solid lines, respectively. Specifically, the MB array shows a broadband valley around the frequency of 0.723 THz, which is usually believed to result from Lorentzian resonance caused by incident light [32]. However, the SRR and LRR arrays produce two ultra-narrow transmission valleys at 0.909 THz and 0.778 THz, respectively, which is usually believed to result from LC resonance caused by incident light [26]. The transmittance peaks 1 and 2 arise from the coupling of the narrowband mode and broadband mode, in which the broadband mode is referred to as bright mode and the narrowband mode as dark mode [43]. The bright and dark modes produce destructive interference at specific frequencies due to their different bandwidths, leading to the EIT effect [14].

**Figure 4:**
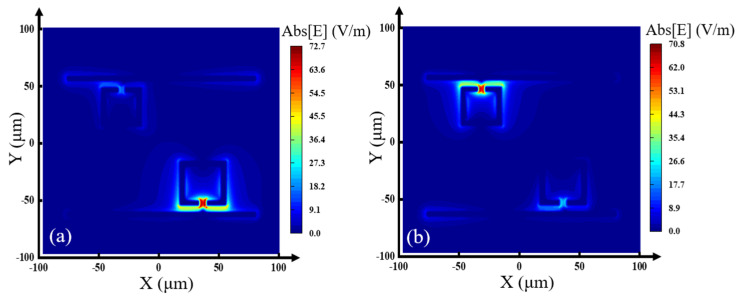
Electric field distribution of the EIT transmission peak at (**a**) 0.73 THz, (**b**) 0.862 THz.

To further explain the formation of the two EIT transmission peaks, as shown in Figure 4a,b, we provided the electric field distribution at 0.73 THz and 0.862 THz, respectively. The EIT effect transmission peak 1 at 0.73 THz is generated from the coupling of the LRR and the MB, while the EIT transmission peak 2 at 0.862 THz results from the coupling of the SRR ring and the MB [32]. The two different couplings produce two EIT transmission peaks in the transmission spectrum, achieving a double-channel effect.

**Figure 5:**
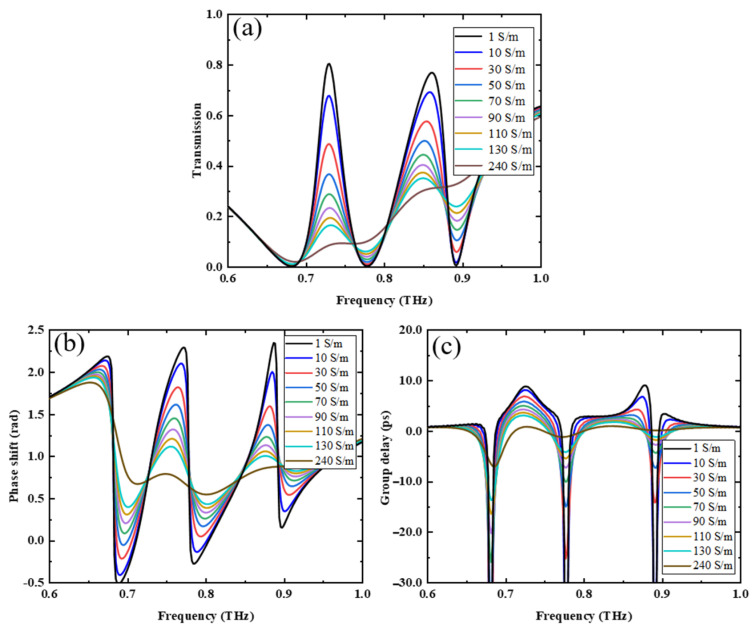
(**a**) Transmission spectrum, (**b**) phase-shift spectrum, (**c**) group delay variation in PSi at different conductivities for a VO_2_ conductivity of 1 S/m.

Secondly, we studied the transmission properties and slow-light effect of the metamaterial when the temperature of VO_2_ is below the phase-change temperature and the pumping light of the PSi is on. In this case, the conductivity of VO_2_ is still 1 S/m and the conductivity of PSi increases from 1 S/m to 240 S/m. The transmission characteristics of the metamaterial are shown in Figure 5a, where we can see that the peak transmittances at 0.73 THz and 0.862 THz are 0.804 and 0.769, respectively, at 1 S/m for PSi. As the conductivity of the PSi increases, the amplitudes of peaks 1 and 2 begin to decrease and the peak frequency at peak 2 begins to redshift. When the conductivity of the PSi reaches 240 S/m, the peak transmittance values at frequencies of 0.73 THz and 0.862 THz are 0.08 and 0.31, respectively, and the transmittance spectral curve becomes an approximate Lorentz curve. The decrease in the transmission is due to the modulation of the dark mode by the increased conductivity of PSi. The PSi is located at the SRR and LRR notch, and an increase in its conductivity causes a decrease in the resonant strength of the SRR and LRR, affecting the coupling of the SRR and LRR with the CW, which in turn affects the amplitude and frequency position of peaks 1 and 2 [26]. The modulation depth of the transmission peak can be expressed as follows [14]:

(3) $$\frac{\Delta T}{T_{0}}=\frac{\left|T_{0}-T_{1}\right|}{T_{0}}$$

where *T*_0_ and *T*_1_ are the transmittance of the PSi conductivity at 1 S/m and 240 S/m, respectively. *T*_0_ and *T*_1_ at 0.73 THz were 0.804 and 0.08, respectively, and *T*_0_ and *T*_1_ at 0.862 THz were 0.769 and 0.31, respectively. Using Equation (3), we can solve for modulation depths of 90.05% and 59.69% at 0.73 THz and 0.862 THz, respectively.

The slow-light effect is an important application of EIT and is often described by the group delay:

(4) $$G=\frac{d\varphi }{d\omega }$$

where φ is the phase shift of EIT and ω=2πf is the angular frequency of EIT [14]. Figure 5b,c show the phase shift and group delay of the EIT effect for different conductivities of PSi in Figure 5a. When the conductivity of PSi is 1 S/m, the phase shift shows steep jumps near the two transmission peaks and achieves group delays of 8.79 ps and 9.04 ps, corresponding to free-space propagation distances of 2.64 mm and 2.71 mm. This means that by controlling the conductivity of the photosensitive silicon, the optical path difference can be varied. The effect can be used in practical applications such as quantum communications and slow optical devices [30]. As the conductivity increases, the phase shift decreases, and group delay gradually diminishes. When the conductivity of the PSi reaches 240 S/m, the phase shift jumps, the group delay properties almost disappear, and the slow-light effect caused by the MB gradually becomes apparent. Thus, the regulation of the double slow-light effect can be achieved by changing the conductivity of the PSi.

Then, at different conductivities of photosensitive silicon, the theoretical fitting of the EIT transmission spectrum is carried out using the two-resonator model expressed by specific equations which are as follows [44]:

(5) $$\begin{array}{cc} {\ddot{x}}_{1}(t)+\gamma_{1}{\dot{x}}_{1}(t)+w_{1}^{2}x_{1}(t)+\Omega^{2}x_{2}(t)= & \frac{Q}{M}E\\ {\ddot{x}}_{2}(t)+\gamma_{2}{\dot{x}}_{2}(t)+w_{2}^{2}x_{2}(t)+\Omega^{2}x_{1}(t)= & \frac{q}{m}E \end{array}$$

(6) $$\chi =\frac{\kappa }{A^{2}B}(\frac{A(B+1)\Omega^{2}+A^{2}((\omega^{2}-w_{2}^{2})+B(\omega^{2}-w_{1}^{2}))}{\Omega^{4}-(\omega^{2}-w_{1}^{2}+i\omega \gamma_{1})(\omega^{2}-w_{2}^{2}+i\omega \gamma_{2})}\;+i\omega \frac{A^{2}\gamma_{2}+B\gamma_{1}}{\Omega^{4}-(\omega^{2}-w_{1}^{2}+i\omega \gamma_{1})(\omega^{2}-w_{2}^{2}+i\omega \gamma_{2})})$$

**Figure 6:**
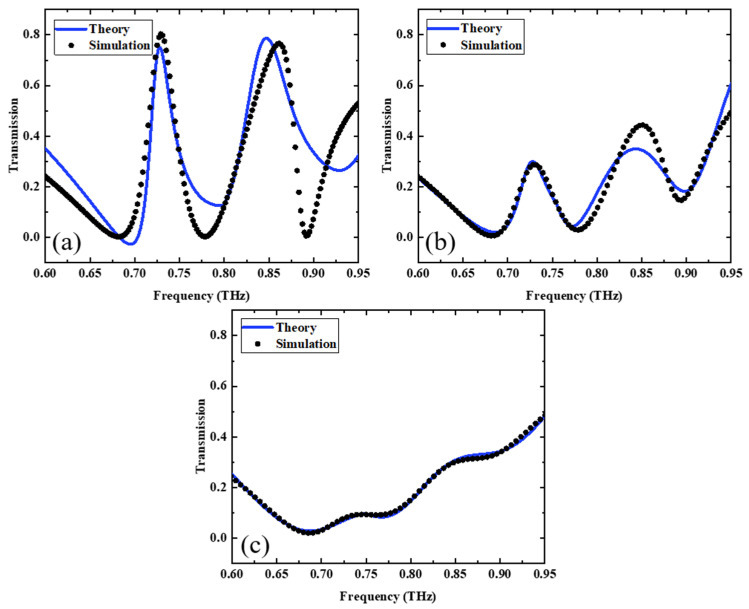
Simulated and theoretical fitted transmission spectra of LRR coupled to MB at photosensitive silicon conductivities of (**a**) 1 S/m, (**b**) 70 S/m, (**c**) 240 S/m.

(7) T=1−lm(χ)

where *x*_1_ and *x*_2_ are the displacement, and *E* is the incident electric field. (*w*_1_, *w*_2_) and ($\gamma_{1}$, $\gamma_{2}$) are the resonant angular frequencies and loss factors of the bright and dark modes, respectively, Ω denotes the coupling strength between the bright and dark modes, and (*Q*, *q*) and (*M*, *m*) denote the effective charge and effective mass, respectively. The dimensionless constants, *A* and *B*, are defined using *A* = *Q*/*q* and *B* = *M*/*m* to describe the relative coupling of the incident light to the bright and dark modes. The linear polarization rate χ can be obtained with Equation (5) and the transmission coefficient T can be derived from theoretical analysis. Figure 6a–c show the theoretical and simulated curves of the EIT transmission spectra using the coupled resonator model when the conductivities of PSi are 1 S/m, 70 S/m, and 240 S/m, respectively. From the figure, it can be seen that the theoretical fitted curves match well with the simulated curves in the EIT functional mode.

**Figure 7:**
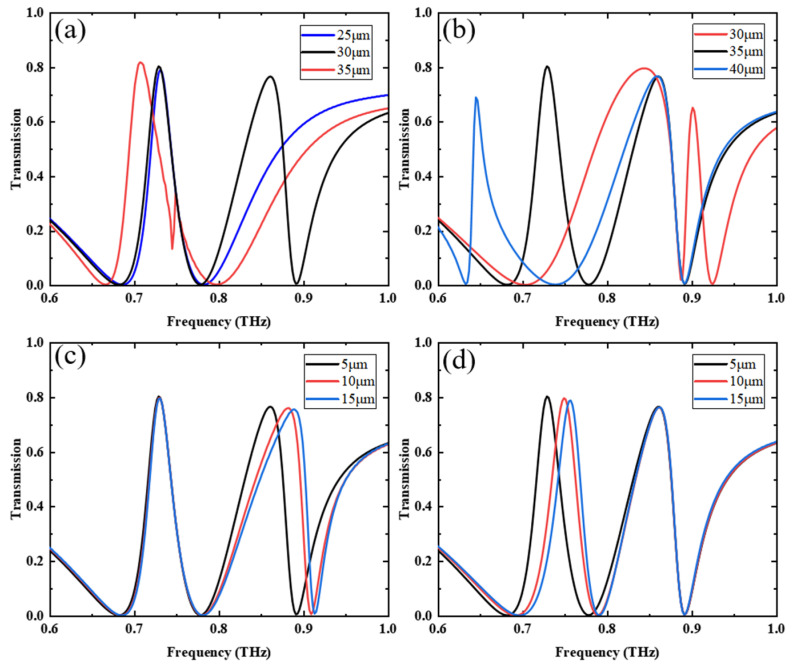
(**a**) Transmission spectrum when LRR size is constant and SRR size is varied, (**b**) transmission spectrum when the SRR size is constant and the LRR size is varied; EIT effect transmission spectra at different (**c**) SRR, (**d**) LRR, and CW distances.

Finally, we studied the size effect of the metamaterial when VO_2_ is below the phase-change temperature and the pumping power of the photosensitive silicon is off. In this case, the conductivity of VO_2_ and photosensitive silicon is 1 S/m, and the results are shown in Figure 7. Figure 7a shows the transmission spectrum for a constant LRR size of 35 μm and SRR sizes of 25 μm, 30 μm, and 35 μm, respectively. As the size of the SRR increases, a redshift occurs in the transmission peak and the two transmission valleys on the right side. Figure 7b shows the transmission spectrum for a constant SRR size of 30 μm and for LRR sizes of 30 μm, 35 μm, and 40 μm, respectively. As the LRR size increases, a redshift occurs in the left transmission peak and the two transmission valleys. The peak transmission on the left side is related to the size of the LRR, while the peak transmission on the right side is related to the size of the SRR. Figure 7c shows the transmission spectra of the SRR at 5 μm, 10 μm, and 15 μm distances from the MB when the LRR is at a constant distance of 5 μm from the MB. As the distance increases, the transmission peak and two transmission valleys on the right show a blueshift. Figure 7d shows the transmission spectra of the LRR with MB distances of 5 μm, 10 μm, and 15 μm when the SRR is at a constant distance of 5 μm from the MB. As the distance increases, the transmission peak and two transmission valleys on the left experience a blueshift. It is further shown that the transmission peak on the left side is related to the LRR and the transmission peak on the right side is related to the SRR. In summary, the dual-channel effect is generated by the coupling of two resonant rings to metal bars.

### 3.2. Absorption

**Figure 8:**
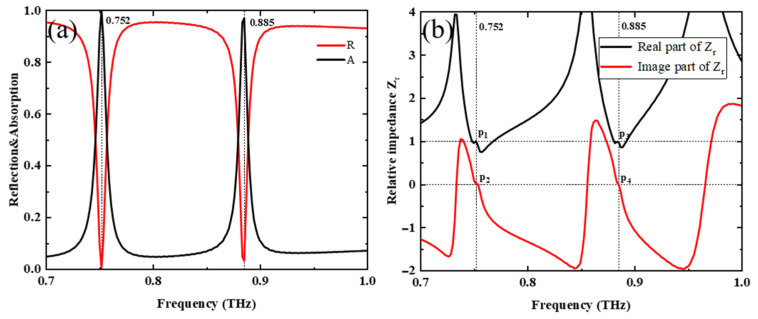
(**a**) Absorption and reflection spectra of metamaterials with PSi conductivity of 1 S/m and VO_2_ conductivity of 2 × 10^5^ S/m, (**b**) schematic diagram of the real and imaginary parts of impedance matching.

When the pumping power of the PSi is off, its conductivity is 1 S/m. When VO_2_ is above the phase-change temperature (87 °C) [42], its conductivity is 2 × 10^5^ S/m, and it is in its metal state. In this case, we studied the absorption properties of the metamaterial. Absorbance can be calculated using *A* = 1 − *R* − T = 1 − ${\left|S_{11}\right|}^{2}$ − ${\left|S_{21}\right|}^{2}$, where *R* stands for reflectance and T for transmittance, and the $S_{11}$ and $S_{21}$ are the reflection and transmission coefficients obtained from the S-parameters [45]. The thickness of VO_2_ film is 200 nm and larger than the terahertz crust depth, which makes terahertz almost impermeable [9], so T = 0, *A* = 1 − *R* = 1 − ${\left|S_{11}\right|}^{2}$. The reflection spectrum was calculated using FDTD, and correspondingly the absorption spectrum was obtained and is shown in Figure 8a, where two absorption peaks are at 0.752 THz and 0.885 THz, respectively.

We studied the origin of the absorption peaks using impedance-matching theory, shown as follows [46,47,48]:

(8) $$A=1-R=1-{\left|\frac{Z-Z_{0}}{Z+Z_{0}}\right|}^{2}=1-{\left|\frac{Z_{r}-1}{Z_{r}+1}\right|}^{2}$$

(9) $$Z_{r}=\pm \sqrt{\frac{(1+S_{11}{)}^{2}-{S_{21}}^{2}}{(1-S_{11}{)}^{2}-{S_{21}}^{2}}}$$

where *Z* is the effective impedance, *Z*_0_ is the free-space impedance, and the relative impedance is expressed as $Z_{r}=Z/Z_{0}$. When $Z_{r}=1$, the absorption rate is 1 and the impedance of the metamaterial matches that of the free space. Figure 8b shows the corresponding impedance plots, where the real part and imaginary part of the relative impedance $Z_{r}$ are shown with black and red lines, respectively. At p_1_ and p_2_, the real and imaginary values of the relative impedance $Z_{r}$ are 1 and 0, respectively, which means impedance matching occurs here. The same effect happens at p_3_ and p_4_, and p_1_, p_2_, p_3_, and p_4_ present evidence of perfect absorption at 0.752 THz and 0.885 THz, respectively.

**Figure 9:**
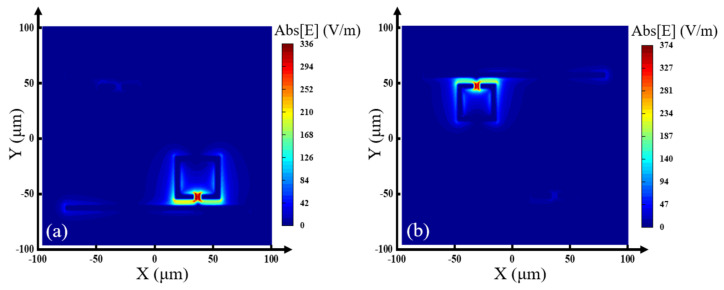
Electric field distribution of the absorption peak at (**a**) 0.752 THz and (**b**) 0.885 THz.

To further explain the formation of the two absorption peaks, we calculated the electric field distribution of the structure at frequencies of 0.752 THz and 0.885 THz, which are shown in Figure 9a and Figure 9b, respectively. From Figure 9a, we can find that the electric field enhancement is mainly distributed in the gap between the LRR and the MB, which means the absorption peak at 0.752 THz mainly originates from the coupling of the LRR to the MB. From Figure 9b, we can see that the electric field enhancement is mainly distributed in the gap between the SRR and the MB, which means the absorption peak at 0.885 THz mainly originates from the coupling of the SRR to the MB. The two different coupling modes produce a double narrow-band absorption peak in the absorption spectrum.

**Figure 10:**
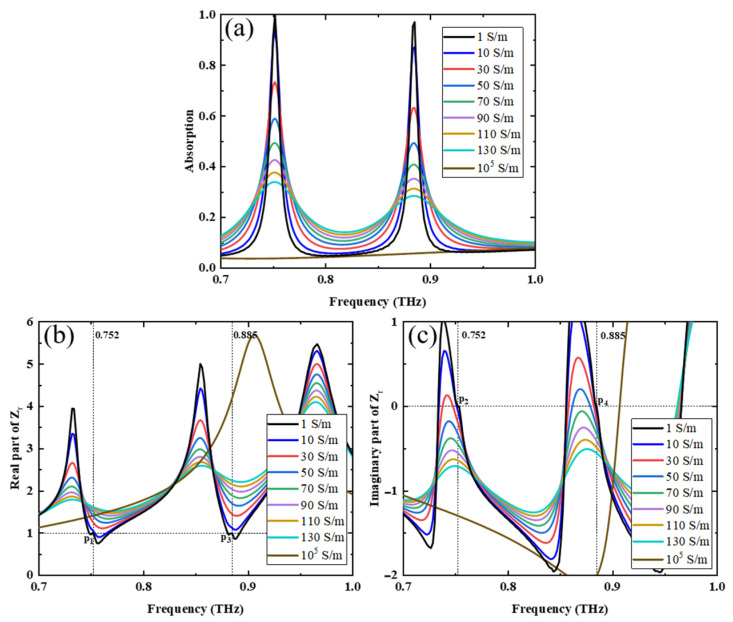
(**a**) Absorption spectra of PSi at different conductivities when the conductivity of VO_2_ is 2 × 10^5^ S/m; (**b**) real and (**c**) imaginary parts of relative Z_r_ for PSi at different conductivities when the conductivity of VO_2_ is 2 × 10^5^ S/m.

In addition, we studied the absorption properties of the metamaterial when VO_2_ is above the phase-change temperature and the pumping power of the PSi is on. In this case, the conductivity of VO_2_ is 2 × 10^5^ S/m and the conductivity of PSi is adjusted from 1 S/m to 10^5^ S/m by changing the intensities of the pump light. Figure 10a shows the change in absorption with the conductivity of PSi. When the conductivity is 1 S/m, the absorption peak is the highest. As the conductivity increases, the absorption peak decreases gradually. When the conductivity is 10^5^ S/m, the absorption peak disappears, which means that the resonance between LRR and SRR and MB disappears. The above simulation results were then analyzed quantitatively using impedance-matching theory. The real and imaginary parts of the relative impedance Zr were calculated using the MATLAB program for different PSi conductivities, and the results are shown in Figure 10b,c. As shown in Figure 10b, as the PSi conductivity increases, the real part of the relative impedance Zr at the peak absorption frequency gradually deviates from 1. Similarly, in Figure 10c, as the PSi conductivity increases, the imaginary part of the relative impedance Zr at the peak absorption frequency gradually deviates from 0. These theoretical data validate the simulation results in Figure 10a, where the absorption peak decreases as the PSi conductivity increases.

In fact, at peak frequency, the conductivity of the PSi located at the SRR and LRR notches affects the impedance match between the metamaterial and the free space. High conductivity causes weak matching and low absorption [9]. Specifically, when the conductivity of PSi was 1 S/m or 10^5^ S/m, the amplitudes of absorption at 0.752 THz and 0.885 THz were 0.998 and 0.97 or 0.036 and 0.055, respectively. So, the modulation depths of the structure can be obtained via Equation (3) to be 96.39% and 94.33% at 0.752 THz and 0.885 THz frequencies, respectively.

**Figure 11:**
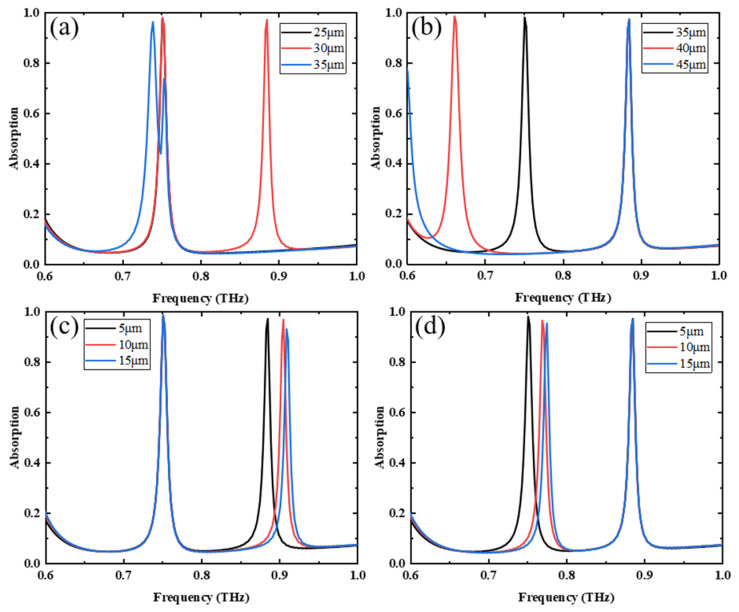
(**a**) Absorption spectrum when LRR size is constant and SRR size varies; (**b**) absorption spectrum when SRR size is constant and LRR size varies; absorption spectra at different (**c**) SRR, (**d**) LRR, and CW distances.

Finally, we studied the size effect of the metamaterial when VO_2_ is above the phase-change temperature and the pumping power of the photosensitive silicon is off. In this case, the conductivity of VO_2_ is 2 × 10^5^ S/m and photosensitive silicon is 1 S/m, and the results are shown in Figure 11. Figure 11a shows the absorption spectra for a constant LRR size of 35 μm and SRR sizes of 25 μm, 30 μm, and 35 μm, respectively. As the SRR size increases, the absorption peak on the right side shows a redshift. Figure 11b shows the absorption spectra of a constant SRR size of 30 μm and 35 μm, 40 μm, and 45 μm LRR sizes, respectively. As the LRR size increases, the absorption peak on the left shows a redshift. Therefore, the absorption peak on the left side is related to the size of the LRR, while the absorption peak on the right side is related to the size of the SRR. Figure 11c shows the absorption spectra of the SRR at 5 μm, 10 μm, and 15 μm from MB when the LRR is kept at a constant distance of 5 μm from the MB. As the distance increases, the absorption peak on the right side shows a blueshift. Figure 11d shows the absorption spectra of the LRR at 5 μm, 10 μm, and 15 μm from the MB when the SRR is kept at a constant distance of 5 μm from the MB. As the distance increases, the absorption peak on the left shows a blueshift. In summary, the double absorption peak is generated by the coupling of the two resonant rings to the metal bar.

For comparison, in Table 1 we list some related work. From the table, we found that the proposed structure has a higher modulation depth for the EIT effect. Moreover, it also has a higher absorption modulation depth.

## 4. Conclusions

In summary, we have designed a dual-function switchable and tunable THz metamaterial by using VO_2_ and PSi. When VO_2_ was in the dielectric state, the THz metamaterial produced a double EIT effect at frequencies of 0.73 THz and 0.862 THz and achieved group delays of 8.79 ps and 9.04 ps. And varying the conductivity of PSi could tune the amplitude of the transmission peak and group delay. When VO_2_ was in the metallic state, the metamaterial produced two perfect absorption peaks at 0.752 THz and 0.885 THz. And the amplitude of the absorption peak could be tuned by varying the conductivity of PSi. The modulation depths of the two absorption peaks could reach 96.39% and 94.33%, respectively. Compared to previous designs, we have achieved a dual-function integration that breaks the limitations of a single function and achieves a higher modulation depth per function, which will provide devices like optical modulators, optical switchers, and photodetectors with higher sensitivity.

## Figures and Tables

**Table 1:** Comparison of designed metamaterials with reported metamaterials.

Reference	Adjustable Material	Maximum Modulation Depth through the Peak (EIT)	Maximum Modulation Depth of Peak Absorption	Maximum Group Delay (ps)
[28]	Graphene	81%	none	3.6
[29]	None	75.58%	none	none
[49]	VO_2_ and graphene	70%	90%	none
[27]	VO_2_	None	78%	none
[50]	STO and graphene	82.46%	none	1.47
[51]	Silicon	61.8%	none	none
This work	Silicon and VO_2_	90.05%	96.39%	9.04

## Data Availability

All content and data are displayed in the manuscript.
